# An Oxygen Delivery Polymer Enhances Seed Germination in a Martian-like Environment

**DOI:** 10.1089/ast.2019.2056

**Published:** 2020-07-08

**Authors:** John G. MacDonald, Karien Rodriguez, Stephen Quirk

**Affiliations:** Kimberly-Clark Corp., Roswell, Georgia.

**Keywords:** Extraterrestrial crops, Biological life support, Colonization, Plant growth.

## Abstract

Critical to the success of establishing a sustainable human presence on Mars is the ability to economically grow crop plants. Several environmental factors make it difficult to fully rely on local resources for agriculture. These include nutrient sparse regolith, low and fluctuating temperatures, a high amount of ultraviolet radiation, and water trapped locally in the form of ice or metal oxides. While the 96% CO_2_ martian atmosphere is ideal to support photosynthesis, high CO_2_ concentrations inhibit germination. An added difficulty is the fact that a vast majority of crop plants require oxygen for germination. Here, we report the production of a polymer-based oxygen delivery system that supports the germination and growth of cress seeds (*Lepidium sativum*) in a martian regolith simulant under a martian atmosphere at 101 kPa. The oxygen-donating system is based on a low-density lightly cross-linked polyacrylate that is foamed and converted into a dry powder. It is lightweight, added in low amounts to regolith simulant, and efficiently donates enough oxygen throughout the volume of hydrated regolith simulant to fully support seed germination and plant growth. Germination rates, plant development, and plant mass are nearly identical for *L. sativum* grown in 100% CO_2_ in the presence of the oxygen-donating lightly cross-linked polyacrylate compared with plants grown in air. The polymer system also serves to protect root structures and better anchors plants in the regolith simulant.

## 1. Introduction

Successful long-term colonization of Mars is dependent on several factors. One of the most important is the ability to sustainably grow plants for food or as part of a biological life support system (Murukesan *et al.*, 2016). However, the environmental conditions on Mars are problematic to say the least for agriculture. Foremost is the fact that martian surface soil (regolith) is devoid of organic material and reactive nitrogen and contains high concentrations of magnesium, aluminum, and other salts that are refractory to plant growth (Foley *et al.*, 2003); more recently, the Curiosity mission has detected the presence of reactive nitrogen in the form of nitrous oxide.

Although not directly usable, martian regolith does contain nutrients needed to sustain plant growth (most notably phosphorous, iron, and potassium), but they are not in a form that can be immediately utilized to sustain plant growth (Arvidson *et al.*, 2014), yet they could be utilized as plant growth resources as needed. Although lunar and martian regolith is rich in metal oxides, the molecular oxygen that is required for germination would have to be extracted from regolith and reintroduced during seed planting. Mars has a reduced gravity environment of 0.38 g, an atmospheric pressure of 0.7 kPa, a high ultraviolet flux, and relatively severe temperature fluctuations (de Vera *et al.*, 2010; Cockell, 2014).

In favor of utilizing more *in situ* environmental components to support large-scale plant growth is the fact that the martian atmosphere is composed of 95.9% carbon dioxide and that experiments have shown that martian regolith simulant can in fact support plant growth (Wamelink *et al.*, 2014). Water may be abundant in the polar regions or in shaded crater microenvironments (Farmer *et al.*, 1976; Nelson, 2015), but again, would have to be relatively near agricultural stations. Still, the possibility of attainable water *in situ* increases the probability of success for large-scale agricultural endeavors.

Most of these environmental variables can be overcome by creating an artificial growth environment on the surface of Mars. For instance, plants can be grown in enclosures that are atmospherically and temperature controlled and that are shielded from radiation. Recreated Earth-like conditions can include soil, soil-like simulants, or hydro-/aero-ponic plant growth systems shipped from Earth. There are difficulties associated with all these approaches; primarily, the amount of material needed for scale and the lack of sustainability of growing plants independent of martian resources. Regardless of the final design of a martian agriculture system (or bioregenerative life support system), it will be critical to directly utilize as many native resources as possible.

Recent studies in which simulant regolith was used indicate that martian regolith may be capable of supporting the growth of some native species of plants (Visscher *et al.*, 2010; Wamelink *et al.*, 2014) directly or perhaps with some form of processing. In addition, myriad synthetic bioengineering has been employed to increase the photosynthetic potential and stress tolerance for organisms incorporated into biological life support systems [for a review, see Llorente *et al.* (2018)]. It has been shown (Murukesan *et al.*, 2016) that cyanobacteria can be grown in a pure CO_2_ environment at various partial pressures.

There do not appear to be any reports in the literature, however, that report the successful germination/growth of crop plants grown in Mars regolith simulant in a pure or nearly pure CO_2_ atmosphere. Finally, it does appear that the level of martian gravity is sufficient for proper root development [for a review, see Kiss (2014)]

It is critical therefore to utilize local resources as much as possible to establish the first generation of plant crops and a robust biological life support system (Perchonok *et al.*, 2012; Verseux *et al.*, 2015). One potential difficulty in relying primarily on Mars's atmosphere and regolith to establish and maintain crop plants is the requirement of oxygen during seed germination. Although some plants are capable of anoxic germination, most notably rice [for a review, see Magneschi and Perata (2009)], most plants have an absolute oxygen requirement for germination. Germination in the genus *Brassica*, for example, is eliminated below 2% O_2_ and is severely limited below 5% O_2_ (Park and Hasentein, 2016).

In general, monocot genera can germinate in lower partial O_2_ pressures than can dicot genera (Alani *et al.*, 1985), but there is still a germination pathway oxygen dependence. The major effect of anoxia/hypoxia in plant germination is an elimination/reduction in mitochondrial respiration [for a review, see Shingaki-Wells *et al.* (2014)] and a switch to fermentation as the sole source of adenosine triphosphate (ATP) production (Kennedy *et al.*, 1992). At the molecular genetic level, there are common transcriptomic responses and species-specific responses when germination transcription patterns are analyzed during anoxia (Narsai and Whelan, 2013).

Seed germination is composed of three phases (Nonogaki, 2006). Phase I is characterized by the initial uptake of water, which plateaus in phase II when embryo growth occurs. During phase II, initial metabolism begins and general physiological mechanisms (*i.e.*, gravitropism) emerge. The germination process ends in phase III, which is marked by radicle emergence and elongation. The effects of hypoxia/anoxia have been shown to occur during phase II (Paul *et al.*, 2017; Zhou *et al.*, 2017). This is the same phase that is affected by temperature extremes (Labouriau, 1978; Bradford, 1986). The oxygen dependence of seed germination is problematic when considering crop agriculture on Mars.

Although crop plants will eventually begin to produce their own oxygen, an initial intervention is required to supply oxygen for germination. Such an intervention is the subject of this article. The issues of the effects of hypoxia and anoxia are also critical for the design and functioning of bioregenerative life support systems and habitats (Paul and Ferl, 2006; Richards *et al.*, 2006).

Besides molecular oxygen, reactive oxygen species (ROS), including singlet oxygen, hydroxyl radical, hydrogen peroxide, and superoxide, have been shown to play important roles in seed germination (Jeevan Kumar *et al.*, 2015; Ray *et al.*, 2016; Singh *et al.*, 2016). Their role in the germination process is to help balance phytohormone ratios (most notably between abscisic acid and gibberellic acid) that govern the start and extent of germination, as well as to muster protein reserves in the seed (Bailly *et al.*, 2008). They are also critical to the regulation of seed dormancy (Oracz *et al.*, 2007; Leymarie *et al.*, 2012). ROS act as signaling molecules during early plant development and are generated through plant redox reactions (most notably photosynthesis and respiration) from molecular oxygen. Thus, oxygen in all forms becomes the critical variable for the success of crop plant utilization on Mars.

The ideal way to sustainably grow crop species on Mars would be to utilize as much of the naturally occurring resources as possible, including regolith, atmosphere, and water. This leaves molecular oxygen as the only required plant growth component not immediately or readily available on Mars. Although it is possible to generate oxygen from regolith metal oxides, this approach may not be feasible; at least initially, for the purpose of seed germination considering the level of crops required to sustain a colony. It may be more feasible to produce oxygen from *in situ* water via electrolysis, which is a relatively low-energy consuming process; however, the crop enclosures would need to be near where the water is located.

This might mean that alternative oxygen-generating scenarios are needed. A possible alternative way to deliver oxygen to seeds at planting would be to add a lightweight oxygen-generating system directly to regolith. This system should be compact and lightweight so that it can be shipped economically from Earth. It should efficiently liberate oxygen so that a minimum of it is needed per seed planted or per volume of regolith planted. It should be inexpensive to produce, and finally, it should not otherwise impair plant development.

We have developed (Soerens *et al.*, 2006) a lightly internally cross-linked oligomeric polyacrylic acid, partially neutralized sodium salt polymer system (lightly cross-linked polyacrylate). It has good water absorption kinetics and capacity to retain and donate water. Furthermore, by adding stoichiometric equivalent amounts of sodium hydroxide and hydrogen peroxide to the water solution of this polyacrylic acid, sodium salt before drying, we produce an oxygen infused foamed solid hydrogel after drying. This foamed matrix can be used to deliver, in a controlled manner, oxygen in the form of gas (MacDonald *et al.*, 2017). We have tested this polymer-based system to see if it can deliver oxygen in the vicinity of a planted seed. The material can either be mixed into the regolith simulant or coated around a seed.

The presence of oxygen in an otherwise anoxic microenvironment specifically supports the germination process. In fact, the oxygenating lightly cross-linked polyacrylate material permits plant growth not only in martian regolith simulant but also in a pressurized martian atmosphere. To the best of our knowledge, this is the first report of successful germination of a crop seed in a combined Mars-like regolith/atmosphere environment.

## 2. Materials and Methods

### 2.1. Lightly cross-linked polyacrylate

Polyacrylic acid, sodium salt (70% neutralized), that is polymerized with methacryloxy-propyl-trimethoxysilane, was obtained from Evonik Corporation (Richmond VA, designated as polymer SR1717). It is a 250 kDa oligomer and was supplied as a 32% (wt/wt) solids in a water solution. It was cured by placing a sample of the liquid in a pan in a convection oven at 55°C for 20–30 min to drive off the water. As the water was removed, the silanol groups on the polymer chain begin to cross-link, forming a transparent lightly cross-linked polyacrylate hydrogel. This was then ground into a powder (Culatti grinder/hammer mill; model DCFH88) fitted with 1 mm sieve.

### 2.2. Lightly cross-linked polyacrylate with encapsulated oxygen

To make SR1717 infused with oxygen, the following procedure was used. To 40 g of the polyacrylic acid, sodium salt, was mixed 30.5 g 2 M sodium hydroxide solution and stirred by hand. Next, 6.8 g of 30% wt/wt hydrogen peroxide in water solution was added and stirred. Samples were then poured into Teflon-coated trays and placed in a 55°C convection oven for 20 min. White foam film samples were produced, which had approximately tripled in volume.

The base reacted with the hydrogen peroxide during heating to form oxygen gas that was trapped by the thickening and cross-linking hydrogel to form bubbles or cells of the foam. The foamed sheets were then ground to yield a 1.0 mm particle size powder. The oxygen contained per gram of foam can be measured by taking a known weight of foam and adding it to a known amount of nitrogen sparged distilled water in a sealed container that is fitted with an oxygen probe (FOXY; Ocean Optics, Dunedin, FL). The release profile as a function of time was recorded directly by computer.

### 2.3. Regolith

The Mars regolith simulant JSC Mars-1A was obtained from SNC Orbitec, Inc. (Madison, WI). This was the same regolith simulant used for other plant growth studies (Kral *et al.*, 2004; Wamelink *et al.*, 2014). The composition of the material had been characterized (Carlton *et al.*, 2014); however, we performed additional analytical determinations for carbon, nitrogen, sulfur (as sulfate), and phosphorous (as phosphate) composition using standard analytical chemistry techniques. Galbraith Laboratories, Inc. performed all analytical procedures (Tables 1 and 2).

**Table 1.** Additional Composition of JSC Mars-1A Regolith Simulant

Element/compound	Amount
Organic carbon	2.6%
Carbon as carbonate	0.03%
Nitrogen	0.32%
Nitrate	7 ppm
Phosphorous as phosphate	<99 ppm
Sulfur as sulfate	<10 ppm

**Table 2.** Particle Size Distribution for JSC Mars-1A Regolith Simulant and the Modified Oxygen-Donating Lightly Cross-Linked Polyacrylate

Particle size range (μm)	JSC Mars-1A regolith simulant	Oxygen-donating lightly cross-linked polyacrylate
<100	5	1
101–200	26	9
201–400	38	13
401–600	19	15
601–800	8	27
801–1000	3	35
>1000	1	0

### 2.4. Measuring oxygen release from lightly cross-linked polyacrylate

Oxygen release from oxygen-generating lightly cross-linked polyacrylate was measured by monitoring the amount of oxygen in nitrogen-rich air upon directly wetting the polyacrylate material (no regolith simulant present). For this, 5 mL of N_2_-saturated deionized water (25°C) was added to 1 g of oxygen-generating lightly cross-linked polyacrylate material. Immediately after, the amount of oxygen in N_2_-rich air space (10 cm^3^) was monitored in 10 s intervals for a total of 15 min with an *in situ* oxygen probe (FOXY; Ocean Optics).

Oxygen content in the regolith (30 cm^3^) was measured upon addition of N_2_-rich deionized water to oxygen-generating lightly cross-linked polyacrylate material mixed with N_2_-rich regolith. Immediately after, the amount of oxygen in various points inside the regolith was measured with the FOXY oxygen probe. To assess the evolved oxygen in the growth chambers (as escaped from the regolith simulant volume and/or by the onset of photosynthesis), an Ocean Optics NeoFox phase fluorometer oxygen probe was equipped with a RedEye oxygen patch. The patch was placed inside a growth chamber and evolved O_2_ measurements were recorded every 2 days.

### 2.5. Seeds and growth conditions

Cress seeds (*Lepidium sativum*) were obtained from Kitazawa Seed Company, Oakland, CA. Growth setup conditions were as follows:

**Table:** 

Planting condition	Planter container volume (cm^3^)	Regolith simulant volume (cm^3^)	Mass of N_2_ saturated water added (g)
A	30	25	14
B	190	125	70
C	525	250	140

The three different container sizes served as a control to see if regolith mass or container volume had any role in germination rate or growth kinetics.

For each of the planting conditions above, four atmospheric/regolith conditions were tested:

**Table:** 

Environmental condition	Atmosphere (101 kPa)	Planting medium
I	Air	Regolith
II	Air	Regolith + lightly cross-linked polyacrylate
III	CO_2_	Regolith + lightly cross-linked polyacrylate
IV	CO_2_	Regolith + oxygen-donating lightly cross-linked polyacrylate

In all cases, only one seed was planted per container. Seeds were buried in each container to a depth of 3 mm and covered with regolith. Twenty-five seeds were planted for each planting/environmental combination (*e.g.*, I-A), hence 75 seeds per environmental condition (*e.g.*, I-A, B, C), and 300 seeds for the entire course of the experiment. The lightly cross-linked polyacrylate material or the lightly cross-linked polyacrylate–oxygen-donating material was mixed with regolith simulant at a 1:50 mass ratio (polyacrylate:regolith), except in experiments where the oxygen-donating polyacrylate was titrated to find the optimum ratio for germination.

All experiments were performed in vacuum desiccators so that the atmosphere could be controlled. Planting containers were placed into the desiccators randomly, but with an attempt to create an even distribution of containers across the desiccator platform. A vacuum pump was used to remove the ambient atmosphere (final pressure 10 kPa). A Mars-like atmosphere, pure CO_2_ (99.5%, NexAir gas purity), at a final pressure of 101 kPa was introduced into the vessel. No attempt was made to calculate vapor pressure introduced into the desiccator environment due to the wet regolith simulant. The vessel was then flushed three times in a similar manner to ensure that the ambient atmosphere was completely exchanged.

The desiccator was placed under a plant growth lamp (Sun Blaster Corp.) that delivered a photosynthetic photon flux density of 153 μmol/[m^2^·s]. The tops of the desiccators were the same distance from the light source (as were the regolith surfaces) to maintain an equal photon flux density. All experiments utilized a 12-h photoperiod. Each day the vessels were rotated 90° to ensure uniform light exposure. Plants were grown at room temperature, 25°C, and growth was monitored for 12 days. A single separate experiment was performed to look at total plant mass and evolved oxygen in the desiccator for a 24-day growth period to assess any CO_2_ toxicity and level of photosynthesis.

During the experiment, seeds were scored for percent germination, time to germination, and plants were scored for time to cotyledon pair emergence and time to third leaf appearance. After the 12-day growth experiment, plants were harvested and the biomass above and below the regolith surface (after removing all regolith from the roots via a water wash and desiccating the plant at 70°C for 12 h) was determined.

An experiment was performed to determine whether the extra sodium contained in the lightly cross-linked polyacrylate could be responsible for the observed germination in a CO_2_ atmosphere. Twenty seeds were planted with and without the addition of 0.1% (w/w) NaCl mixed with the regolith simulant, regolith simulant/lightly cross-linked polyacrylate, or regolith simulant/oxygen-donating lightly cross-linked polyacrylate. Percent germination, germination lag time, and the time to reach 50% germination were calculated.

A final growth experiment was conducted to extend the growth period from 12 to 24 days to assess full plant growth potential. Two conditions were selected for this experiment, II-A (regolith simulant and lightly cross-linked polyacrylate in air) and IV-A (regolith simulant and oxygen-donating lightly cross-linked polyacrylate in CO_2_).

All chemicals were obtained from Sigma–Aldrich Chemical Company (Milwaukee, WI) unless otherwise stated. Gasses were from NexAir Corp (Memphis, TN).

### 2.6. Statistical analysis

Mass differences between planting conditions within a given environmental combination (*e.g.*, I-A vs. I-B vs. I-C) were calculated in the statistical package R (R Core Team, 2014) and are presented as mean ± standard deviation. Mass differences between the environmental conditions (*e.g.*, I-A, -B, -C vs. II-A, -B, -C) are presented as the grand mean ± standard deviation. A Kolmogorov–Smirnov test or the Wilcoxon rank-sum test (in R) was used to calculate statistical significance between the experimental populations.

## 3. Results

### 3.1. Synthesis of lightly cross-linked polyacrylates

**FIG. 1.**
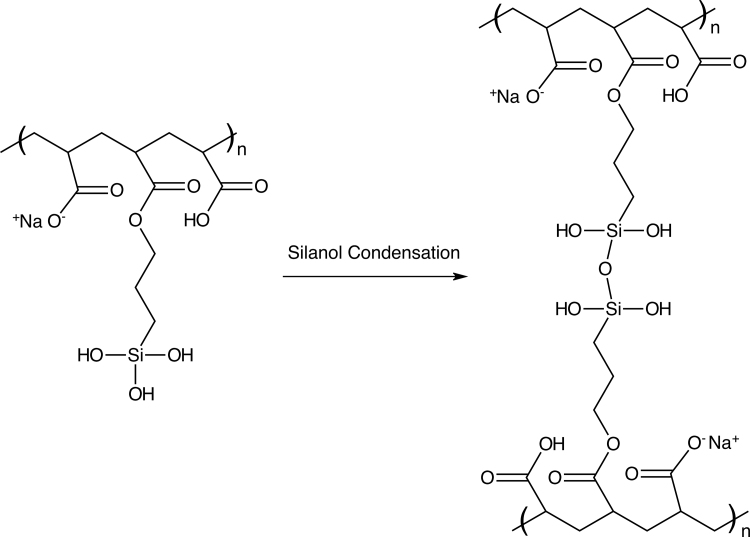
Chemical structure of the lightly cross-linked polyacrylate material. Shown is the structure of the polymer repeating unit and the nature of the siloxy cross-link.

The synthesis of lightly cross-linked polyacrylate and the oxygen-donating polyacrylate was a straightforward process. The chemical structure of the monomer and the lightly cross-linked product are shown in Fig. 1. Typically, 40 g of the modified polyacrylic acid sodium salt in water solution yielded 12.8 g of dried powder. The dried lightly cross-linked polyacrylate powder was easily distributed throughout the martian regolith by simple hand mixing of the powders with a spatula. Similarly, the liquid-modified polyacrylic acid, sodium salt, sodium hydroxide, and hydrogen peroxide mixture yielded 13.0 g of dried powder. Since the density of this foamed powder being significantly less, it was larger in volume for the same weight.

**FIG. 2.**
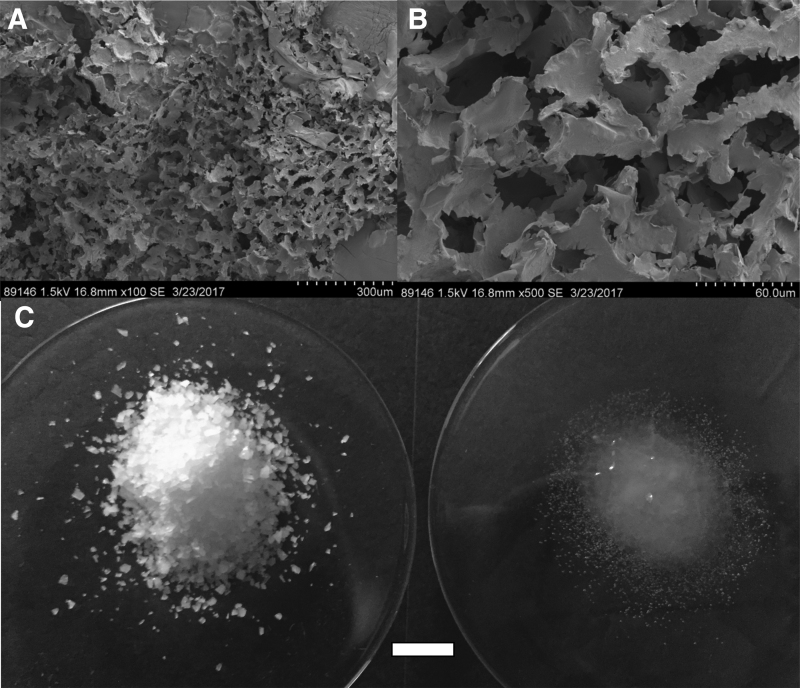
Physical structure of the oxygen-donating lightly cross-linked polyacrylate material. Shown are SEM images of the powdered material **(A)** as well as the bulk powder in the unhydrated **(B)** and fully hydrated **(C)** state. SEM, scanning electron micrograph.

The physical structure of the oxygen-donating lightly cross-linked polyacrylate powder is shown in Fig. 2. It displays typical hydrogel-like structure, both under extreme magnification as well as when hydrated. The oxygen-generating material swelled approximately twice as fast as the nonoxygen-generating lightly cross-linked polyacrylate (data not shown). This is most likely due to thinner walls in the oxygen-generating hydrogel foam that allows for faster water penetration into the voided spaces. When water was applied to the containers containing regolith simulant and lightly cross-linked polyacrylic acid–sodium salt mixture, the volume increased ∼20% due to the lightly cross-linked polyacrylate swelling on absorbing water.

**FIG. 3.**
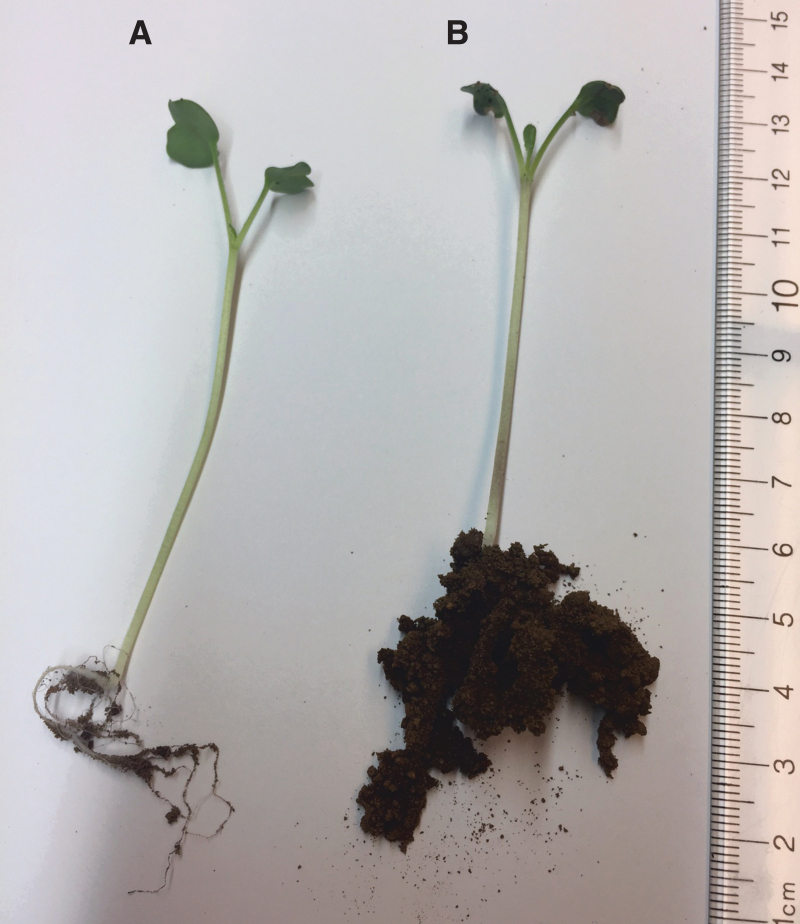
The oxygen-donating lightly cross-linked polyacrylate material acts as a growth matrix to anchor root structure. A 12-day *Lepidium sativum* plant was gently pulled from a regolith only growth container **(A)** or a growth container containing a mixture of regolith and of oxygen-donating lightly cross-linked polyacrylate at a ratio of 50:1 **(B)** and gently shaken to remove any regolith that is loosely associated with plant root structures.

Examination of the roots at the end of the seedling experiments at day 12 clearly showed that the material acts as a water reservoir, as the roots grew through and around the swollen particles. The addition of the material may also serve to loosen/destabilize regolith packing so as to allow stronger root formation. Figure 3 shows how hydrogel/regolith simulant formed a stable structure with the root mass relative to regolith simulant alone.

### 3.2. Composition of regolith simulant

Additional analytical chemistry determinations were conducted to further characterize the JSC Mars-1A regolith simulant. Specifically, determinations were made to assess total carbon, metabolic nitrogen, phosphate, and sulfate content. Although the mineral content of the regolith simulant has been reported (Carlton *et al.*, 2014), these additional determinations have not been previously reported and are shown in Table 1. The regolith simulant does in fact contain 2.6% organic carbon content, which is apparently enough to foster plant growth in our experiments (12- or 24-day). The relatively low nitrogen content, 0.32%, is in a form that is metabolically accessible to the seedlings as it is the only source of metabolic nitrogen in the system under study.

The pH of hydrated regolith simulant was 6.5. The pH decreased to 6.0 when lightly cross-linked polyacrylate is added and rose to 7.0 in the presence of the oxygen-donating lightly cross-linked polyacrylate. Analytical chemistry determinations for regolith simulant in the presence of the lightly cross-linked oxygen-donating polymer were problematic due to the variability in uniformly taking samples that had the same amount of polymer particles. However, when a known amount of polymer (10% w/v) was spiked into a regolith analytical sample, the silicon content of JSC Mars-1A increased by 8% (35–43%) and the sodium content increased by 7% (2–9%).

The particle size distributions (obtained via sieving) of the regolith simulant and the lightly cross-linked oxygen-donating polyacrylate are shown in Table 2. The relative particle size of the polymer did not affect germination results (data not shown), and for all experiments, a mixture of all size classes below 1 mm in diameter was utilized. However, in general, smaller diameter particles were easier to uniformly mix with regolith simulant.

### 3.3. Oxygen-donating kinetics

Modified lightly cross-linked polyacrylate is capable of liberating a significant amount of gaseous oxygen over time. The release kinetics as measured by the *in situ* oxygen probe shows that typically the oxygen-generating lightly cross-linked polyacrylate can release 4.9 mg O_2_/g material, although there is a degree of batch-to-batch variation (±1.1 mg O_2_/g material). The amount of released oxygen was also dependent on the average diameter of the powder particles produced by milling, with larger particles having a slower release kinetics (data not shown) as may be expected. No attempts were made in this work to completely characterize the modulation of oxygen release as a function of material diameter, but release rate could certainly be tailored for the oxygen requirements of a given plant species.

**FIG. 4.**
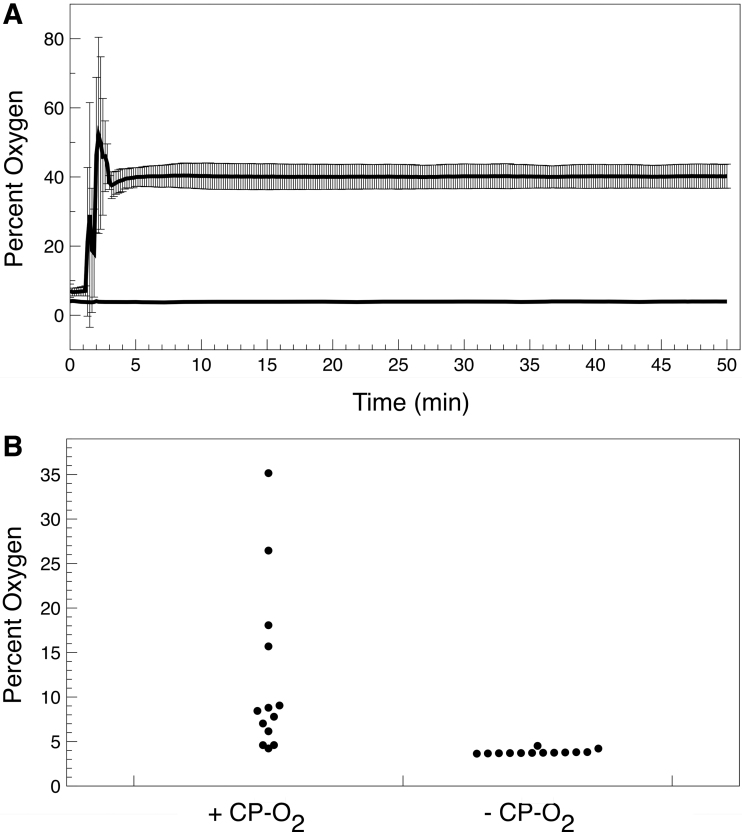
Oxygen release profile. **(A)** Oxygen release kinetics from 1 g of the oxygen-donating lightly cross-linked polyacrylate into an air space of 10 cm^3^ when wet, measured as percent oxygen by the *in situ* probe (closed circles). Open circles are control measurements in the absence of the oxygen-donating lightly cross-linked polyacrylate. **(B)** Distribution of oxygen content in a regolith simulant growth container without (−) or with (+) the oxygen-donating lightly cross-linked polyacrylate. Thirteen random sample locations were probed throughout the regolith container volume.

The oxygen release profile by the material into a nitrogen airspace and into a Mars regolith simulant/sparged water growth container is shown in Fig. 4. This represents the average release profile for a typical sample of polymer representing the entire size distribution. Upon wetting, the material expands and gaseous oxygen was immediately released into the environment (Fig. 4A). Percent oxygen in a nitrogen atmosphere (10 cm^3^) increases from background to 70% within the first 2 min and reached an equilibrium value of 15% within 5 min. The initial increase and subsequent decrease in the percent oxygen level were attributed to pure oxygen traversing the oxygen probe before equilibrium in the measurement vessel air space is achieved.

This experiment simply measured the total possible release of O_2_ from the material when fully and instantaneously saturated with water in the absence of regolith simulant. The release rate when the material is in combination with regolith simulant will be different. When the oxygen probe was placed directly into a container of regolith simulant/oxygen-donating lightly cross-linked polyacrylate, it began to detect the presence of oxygen above background immediately upon wetting. Since the distribution of the oxygen-donating material within the regolith simulant volume was unknown, the oxygen values recorded are presented as a distribution (Fig. 4B), that is each point represents the O_2_ concentration in a random position within the container volume.

The measurement represents oxygen being released into the regolith from multiple locations inside the growth container. The distribution showed overall transient pre-equilibrium oxygen concentrations in the regolith simulant. The release of oxygen is slower in the regolith simulant due to the time it takes for the gas to diffuse to the location of the probe.

### 3.4. Growth of *L. sativum* in martian regolith simulant and various atmospheres

#### 3.4.1. Growth in Earth atmosphere

**FIG. 5.**
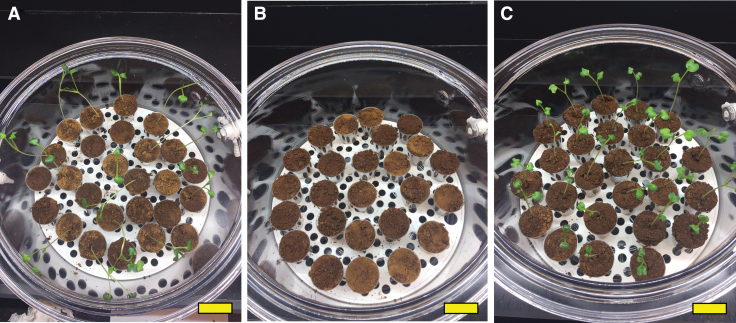
Growth chamber configuration and typical planting results. *Lepidium sativum* seeds were planted in JSC Mars-1A regolith simulant in single containers, which were placed into a vacuum bell jar. Growth conditions were regolith simulant in air **(A)**, regolith simulant supplemented with lightly cross-linked polyacrylate (50:1) in 100% CO_2_ **(B)**, and regolith simulant supplemented with oxygen-generating lightly cross-linked polyacrylate (50:1) in 100% CO_2_ **(C)**. Percent germination is 16/25 **(A)**, 0/25 **(B)**, and 25/25 **(C)**. Atmosphere is controlled via the gas port that can be seen on the right side of the bell jar. The yellow size bar is 4.0 cm.

**FIG. 6.**
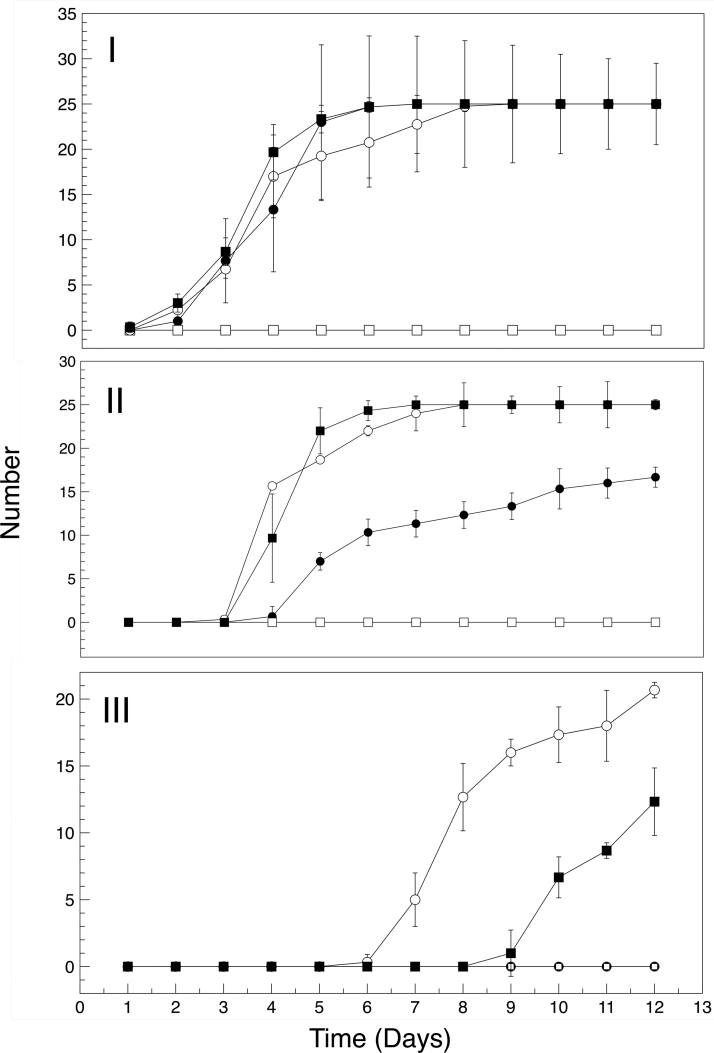
Germination and growth kinetics for *Lepidium sativum* in regolith simulant under various atmospheric conditions. Closed circles, regolith simulant in air; open circles, regolith simulant with lightly cross-linked polyacrylate (50:1) in air; open squares, regolith simulant with lightly cross-linked polyacrylate (50:1) in CO_2_; closed squares, regolith simulant with oxygen-donating lightly cross-linked polyacrylate (1:50) in CO_2_. **(I)** Plumule formation; **(II)** cotyledon pair emergence; **(III)** third leaf appearance. Note that the *y*-axes have different ranges for data visualization clarity. Also note in **(III)**, the open squares and closed circles lines are coincident.

The vacuum desiccator chamber setup provided an efficient means of controlling the atmosphere for the *L. sativum* growth experiments. Figure 5 shows typical planting regimen and results. Mars regolith simulant alone can support the germination and limited growth of *L. sativum* seeds in regular Earth air atmosphere. There was no statistical difference between growth in the three container sizes as measured by the Wilcoxon rank-sum test. All 75 seeds germinated under this condition (I-A, B, C, Table 3 and Fig. 6I, closed circles), but only 15 of the planted seeds continued growth to the stage where leaves emerged (Fig. 6II, closed circles). None of the 15 progressed to third leaf development (Fig. 6III, closed circles). The regolith simulant, even with 2.6% organic carbon and 0.32% metabolic nitrogen, is a poor growth medium.

**Table 3.** Germination Parameters of *Lepidium sativum* in Mars Regolith Simulant

Environmental condition^a^	Planting condition^a^	Germination (%)	Lag*^b^*time (days)	T_50_^c^
Regolith^d^ Air (I)	A	100	1	3.5
	B	100	2	4.5
	C	100	1	3.5
Regolith+CP^e^ Air (II)	A	100	1	3.3
	B	100	1	3.3
	C	100	1	4.0
Regolith+CP	A	0	—	—
	B	0	—	—
	C	0	—	—
CO_2_ (III)			—	—
Regolith+CP-O_2_^f^ CO_2_ (IV)	A	100	1	3.2
	B	100	1	3.1
	C	100	1	3.5

^a^ Each experiment was performed in a different volume planting container with 25 seeds each under different atmospheric/regolith conditions (see Section 2).

^b^ Elapsed days until first germination event.

^c^ Time to reach 50% germination.

^d^ JSC Mars-1A regolith simulant.

^e^ Lightly cross-linked polyacrylate.

^f^ Oxygen-donating lightly cross-linked polyacrylate.

In contrast, all seeds (75/75) planted in regolith simulant containing the lightly cross-linked polyacrylate material (at a 50:1 mass ratio) germinated and continued to grow as healthy plants for the entire length of the experiment (II, A, B, C; Table 3 and Fig. 6, open circles). Figure 6 shows germination and growth development kinetics for all seeds grown in martian regolith simulant with and without the addition of lightly cross-linked polyacrylate. In air (Fig. 6I), there was not a significant difference in initial germination rates (as measured by the emergence of plumule). There was a slight 1-day effect seen at day 4, that indicates that the addition of the lightly cross-linked polyacrylate achieves faster total germination outcomes compared with the control regolith only.

After plumule emergence, the next identifiable stage in *L. sativum* plant development, is the emergence of the cotyledon pair. In air (Fig. 6II), only 60% of seeds developed a cotyledon pair in plain regolith simulant compared with 100% of seeds grown in regolith simulant in the presence of the lightly cross-linked polyacrylate. Cotyledon pair formation was also greatly accelerated. The effects of the addition of lightly cross-linked polyacrylate can be seen in the final stage of *L. sativum* growth, that is, the formation of the third leaf. No plants grown in plain regolith simulant progressed to third leaf development, whereas 84% (63/75) of plants grown in supplemented regolith simulant progressed to this stage by the end of the 12-day growth experiment (Fig. 6III).

**FIG. 7.**
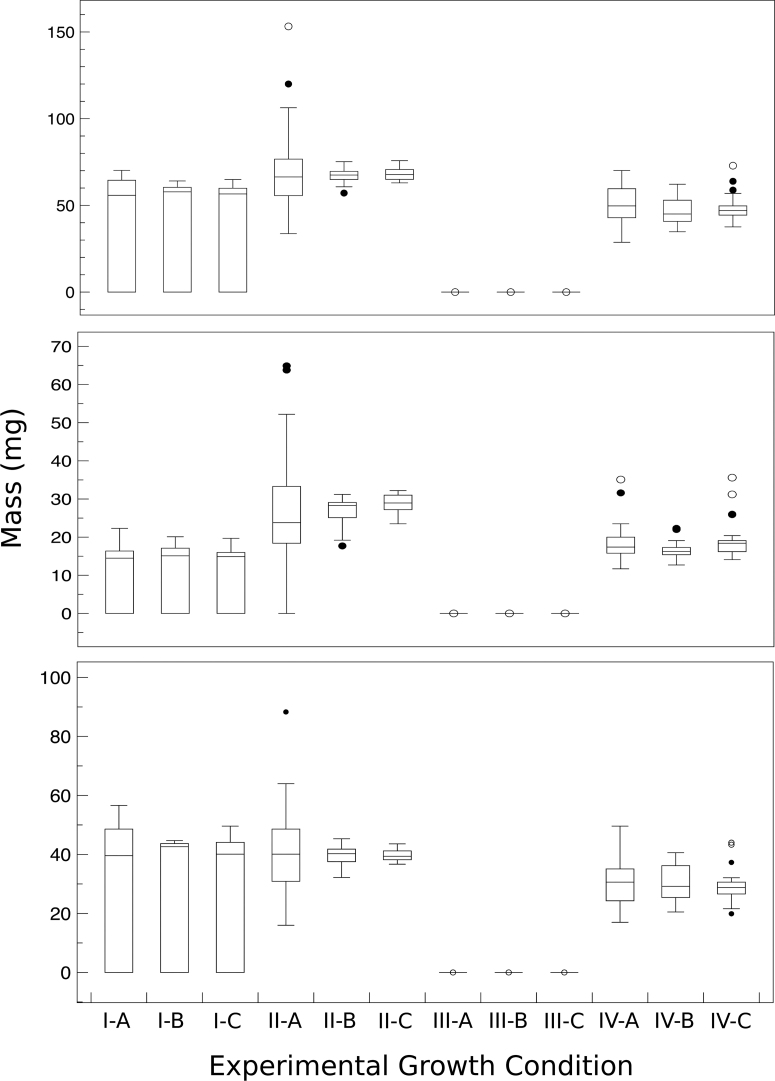
Plant mass as a function of growth condition. Total mass (top panel) of *L. sativum* plants, mass of plant structures above the regolith simulant surface (middle panel), and mass of plant structures below the regolith simulant surface (bottom panel). All *L. sativum* plants were measured post 12 days growth. Growth conditions were as follows: Regolith simulant alone in air **(I)** where A, B, and C refer to the three container sizes, Regolith simulant with lightly cross-linked polyacrylate in air **(II)**, Regolith simulant with lightly cross-linked polyacrylate in CO_2_ **(III)**, and Regolith simulant with oxygen donating lightly cross-linked polyacrylate in CO_2_ **(IV)**. Note that the y-axes have different ranges for data visualization clarity.

Germination and growth parameters are shown in Table 3. There was a measurable statistical difference (using the Kolmogorov–Smirnov two-sample test) between the 12-day total plant mass distribution when *L. sativum* was grown in regolith or regolith/lightly cross-linked polyacrylate (Fig. 7I). The significance level was *p* = 0.002. Similar distribution significances were calculated between the average mass of structures above (Fig. 7II; *p* = 0.001) or below (Fig. 7III; *p* = 0.006) the regolith simulant surface. All masses are given in Table 4. These latter effects are primarily due to the observation that only 60% of plain regolith seeds grew significantly after plumule emergence compared with 100% of seeds planted in regolith supplemented with lightly cross-linked polyacrylate progressing to cotyledon pair emergence and 84% proceeding to the formation of a third leaf.

**Table 4.** Growth of *Lepidium sativum* in Mars Regolith Simulant

Environmental condition	Planting condition	Full growth (%)	Mean*^a^*root mass mg (±SD)	Mean shoot mass mg (±SD)	Mean total mass mg (±SD)
Regolith^b^ Air (I)	A^c^	60	46.0 (6.9)	16.4 (2.9)	62.4 (5.9)
	B	64	43.0 (1.5)	16.3 (2.0)	59.3 (3.0)
	C	60	43.5 (2.7)	16.1 (6.4)	59.6 (2.8)
	Grand mean	61.3	44.1 (8.3)	16.3 (2.3)	60.4 (8.3)
Regolith+CP^d^ Air (II)	A	100	41.3 (15.3)	29.2 (14.7)	70.5 (27.1)
	B	100	39.6 (3.7)	27.4 (3.2)	67.1 (4.2)
	C	100	39.6 (1.8)	28.4 (2.5)	68.0 (3.5)
	Grand mean	100	40.2 (9.3)	28.4 (8.9)	68.5 (16.2)
Regolith+CP CO_2_ (III)	A	0	0.0	0.0	0.0
	B	0	0.0	0.0	0.0
	C	0	0.0	0.0	0.0
	Grand mean	0	0.0	0.0	0.0
Regolith+CP-O_2_^e^ CO_2_ (IV)	A	100	31.4 (9.0)	18.5 (5.3)	49.8 (1.1)
	B	100	29.9 (5.9)	17.0 (2.9)	46.9 (7.7)
	C	100	29.1 (4.9)	19.1 (4.9)	48.2 (8.0)
	Grand mean	100	30.1 (7.0)	18.2 (4.5)	48.3 (9.0)

^a^ Means calculated based only on seeds that germinated and developed during the course of the experiment.

^b^ JSC Mars-1A regolith simulant.

^c^ Each experiment was performed in a different volume planting container with 25 seeds each (see Section 2).

^d^ Lightly cross-linked polyacrylate.

^e^ Oxygen-donating lightly cross-linked polyacrylate.

#### 3.4.2. Growth in a Mars-like atmosphere

To test whether plants could germinate and grow in the regolith simulant/oxygen-donating lightly cross-linked polyacrylate material under a Mars-like atmosphere (although at an Earth pressure of 101 kPa), 100% CO_2_ was introduced in the growth chamber. In a pure carbon dioxide atmosphere (at a pressure of 101 kPa), no *L. sativum* seeds were able to germinate in plain regolith simulant (data not shown) or in regolith simulant supplemented with the lightly cross-linked polyacrylate material (at a 50:1 mass ratio). However, when regolith simulant is mixed with the oxygen-donating lightly cross-linked polyacrylate, *L. sativum* seeds were fully able to germinate and to develop into 12-day-old plants with leaves.

Emergence of plumule and cotyledon pairs in a CO_2_ atmosphere with oxygen-donating lightly cross-linked polyacrylate supplemented regolith simulant was similar to the emergence patterns seen in air (Fig. 6I and II). Third leaf formation was delayed relative to what was seen in air (day 10 vs. day 7), and only 40% (30/75) of plants progressed to the third leaf stage within the 12-day growth experiment (Fig. 6III). Germination and growth parameters are shown in Table 3. Plant masses (and distribution of masses above and below the surface) for *L. sativum* grown in a 100% CO_2_ atmosphere in the presence of oxygen-donating lightly cross-linked polyacrylate were similar to plants grown in air (Table 4 and Fig. 7).

Hence, the main characteristic of *L. sativum* grown in a pure carbon dioxide atmosphere is delayed third leaf development. In air, the lightly cross-linked polyacrylate promoted full plant growth and development compared with plain regolith simulant. In a CO_2_ atmosphere, the oxygen-donating lightly cross-linked polyacrylate material promoted full plant growth and development by providing the oxygen that is required for seed germination. In the absence of the oxygen infusion into the regolith simulant, no seeds germinated (Figs. 6 and 7; Tables 3 and 4). There was no statistical difference between growth in the three container sizes as measured by the Wilcoxon rank-sum test.

There is also the possibility that low amounts of sodium may positively affect the ability of the seeds to germinate and grow (Julkowska and Testerink, 2015), although higher sodium levels are toxic. Such additional sodium may be imparted into the regolith environment from the Na^+^ ion associated with the lightly cross-linked polyacrylate material. To test this, an additional experimental series was undertaken with 20 seeds in each of the environmental growth conditions, with and without the addition of 0.1% (w/w) NaCl. Table 5 indicates that the addition of exogenous sodium had no effect on germination rates or kinetics.

**Table 5.** Effect of Added Sodium to Growth Under a CO_2_ Atmosphere

Growth condition	Experiment^a^	Germination (%)	Lag*^a^*time (days)	T_50_^b^
Regolith	1	0	—	—
	2	0	—	—
	3	0	—	—
Regolith+Na^+^	1	0	—	—
	2	0	—	—
	3	0	—	—
Regolith^c^+CP-O_2_^d^	1	100	2	4.0
	2	100	2	4.0
	3	100	1	5.0
Regolith+CP-O_2_+Na^+^	1	100	2	3.5
	2	100	2	3.0
	3	100	2	4.0
Regolith+CP^e^	1	0	—	—
	2	0	—	—
	3	0	—	—
Regolith+CP+Na^+^	1	0	—	—
	2	0	—	—
	3	0	—	—

^a^ Twenty seeds were planted per experiment. Growth was monitored for 12 days.

^b^ Time to reach 50% germination.

^c^ JSC Mars-1A regolith simulant.

^d^ Oxygen-donating lightly cross-linked polyacrylate.

^e^ Lightly cross-linked polyacrylate.

Hence, the ability of *L. sativum* seeds to germinate and grow in a regolith/CO_2_ environment was due to the presence of the oxygen-liberating cross-linked polyacrylate's oxygen-donating function and not the fact that it is a sodium salt. The overall observation of germination and growth in a pure CO_2_/regolith condition was not a function of container size/volume or added sodium. It was simply due to the availability of molecular oxygen to support germination.

#### 3.4.3. Optimal concentration of oxygen-donating lightly cross-linked polyacrylate

**FIG. 8.**
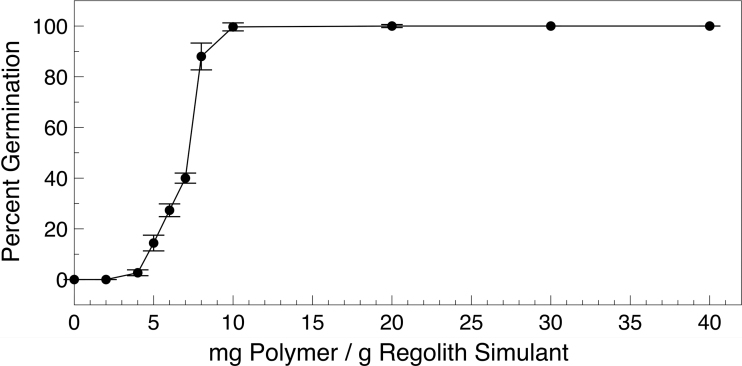
Optimal oxygen level to support germination. Optimal concentration of oxygen-donating lightly cross-linked polyacrylate added to JSC Mars-1A regolith simulant. Plotted is the percent germination of *Lepidium sativum* seeds in 25 g regolith simulant with various amounts of oxygen-donating lightly cross-linked polyacrylate added. Growth was in a 99.5% CO_2_ atmosphere at 101 kPa.

Initial experiments utilizing the oxygen-donating lightly cross-linked polyacrylate material were performed at a mass ratio of 1:50, where 500 mg of the polymer powder was added to 25 g of regolith simulant, for example, in container size 1. To determine the optimal amount to add to the plantings, a titration was performed. When various amounts of the polymer powder, from 50 mg to 1.0 g, were added to regolith simulant in the standard growth regimen, full germination was observed at additions as low as 200 mg (1:125 input ratio). Only 30% of the *L. sativum* seeds germinated at a loading of 150 mg (1:167 input ratio). Germination was fully abolished when 100 mg (1:250 input ratio) or less oxygen-donating lightly cross-linked polyacrylate was added to the Mars regolith simulant. This is summarized in Fig. 8.

All germinated seeds developed into 12-day plants with leaves at rates identical to the data presented in Fig. 6 for the 500 mg (1:50 input ratio; data not shown). Hence the optimal amount of oxygen-donating lightly cross-linked polyacrylate was in the range of 1:125 (polymer powder to regolith simulant). At this concentration, a total of ∼58 ppm of oxygen would be produced per container. This experiment was performed with all growth containers (each containing various amounts of oxygen-donating lightly cross-linked polyacrylate in the regolith simulant) in a single vacuum desiccator at the same time.

The amount of oxygen given off into the desiccator chamber from the regolith simulant surface or from the onset of photosynthesis was insufficient to promote germination of *L. sativum* seeds in containers with ≤100 mg of oxygen-donating lightly cross-linked polyacrylate. Therefore, it was the local concentration of oxygen proximal to the buried seed that is driving germination and not any other factors (*e.g.*, regolith/container volume or added sodium). This local concentration was driven by the ratio of oxygen-generating polymer to regolith.

#### 3.4.4. Extended growth period

**FIG. 9.**
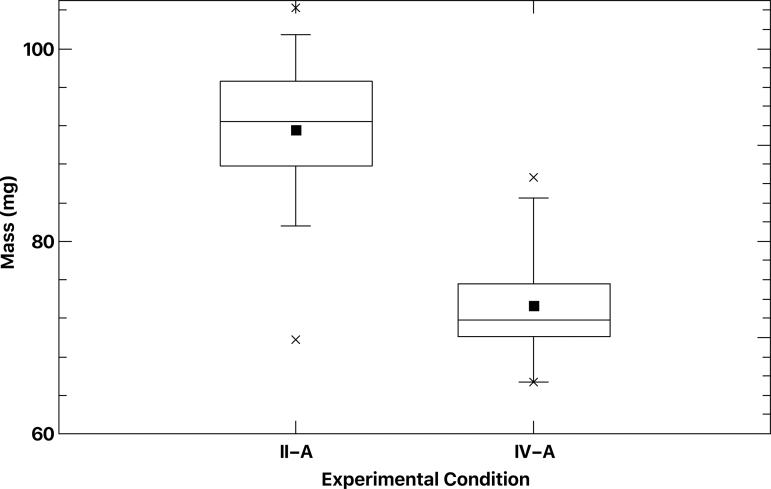
Total *L. sativum* plant mass post 24-day growth. Twenty plants were grown in air/regolith simulant/lightly crosslinked polyacrylate (experimental condition II-A), or in CO_2_/regolith simulant/oxygen donating lightly crosslinked polyacrylate (experimental condition IV-A).

A single growth experiment was conducted to assess whether *L. sativum* plants would continue to grow after the 12-day period utilized to measure germination through third leaf emergence. Twenty plants were grown under experimental condition II-A and 20 plants under experimental condition IV-A. Growth progressed for 24 days at which time plants were harvested and treated as mentioned in Section 2. Plants grown in an air atmosphere in regolith simulant and lightly cross-linked polyacrylate material (condition II-A) increased average total mass from 68.15 ± 16 to 91.5 ± 8 mg/plant, as shown in Fig. 9 (26% increase). Plants grown in a CO_2_ atmosphere in regolith simulant and oxygen-donating lightly cross-linked polyacrylate material (condition IV-A) increased average total mass from 48.3 ± 9 to 72.9 ± 6 mg/plant, as shown in Fig. 8 (33% increase).

These results also indicated that growth in a primarily CO_2_ atmosphere for 24 days retards *L. sativum* mass development by 20.3% (72.9/91.5 mg). Interestingly, at 12 days of growth, the difference between the two plants was more pronounced at 29.1% (48.3/68.15 mg).

#### 3.4.5. Measuring evolved oxygen and the onset of photosynthesis

**FIG. 10.**
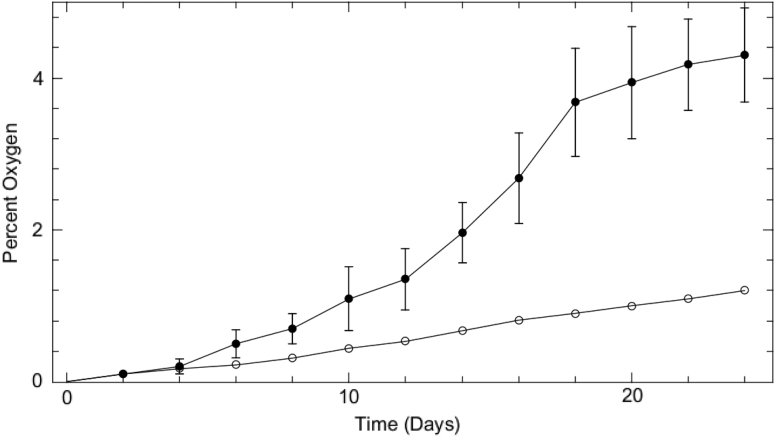
Evolution of oxygen into a growth chamber. Oxygen concentration was measured every 2 days over the course of a 24-day period with an *in situ* oxygen probe. Evolved oxygen from regolith simulant in the absence of *Lepidium sativum* seeds/plants (open circles) and in the presence of *L. sativum* seeds/plants (closed circles) is shown.

An experiment was performed to measure the amount of oxygen released into the volume of a vacuum desiccator chamber (under experimental condition IV-A) over the course of a 24-day growth period. The NeoFox phase fluorometer oxygen probe equipped with a RedEye oxygen patch was utilized to probe O_2_ levels. The patch was placed inside the transparent lid of the vacuum desiccator in direct contact with the atmosphere and was interrogated every 2 days. In the absence of seeds/plants, there was a slow linear increase in the percent of oxygen in the chamber over the 24-day period (Fig. 10, open circles). This represented the amount of O_2_ released from the regolith simulant. The level rises from zero to 1.2%. In the presence of *L. sativum* (Fig. 10, closed circles), the O_2_ level linearly and slowly rises along with the control until day 4.

There was a marked increase in the slope of the O_2_ release line from day 6, which corresponds to cotyledon pair emergence (Fig. 6II), and another increase in the slope of O_2_ release line at day 12, which corresponds to third leaf emergence. This experiment illustrated that sufficient O_2_ is released from the oxygen-donating lightly cross-linked polyacrylate material to fully support germination and to support plant growth through to a point where *L. sativum* can supply its own oxygen. All data discussed in this paper is available in the Supplemental data section.

## 4. Discussion

Variables in the ability of plants to grow in non-Earth environments include adaptation to microgravity environments (Wolverton and Kiss, 2009; Wheeler, 2010; Kordyum, 2014; Jost *et al.*, 2015; Kordyum, and Chapman, 2017; Böhmer and Schleiff, 2019; Vandenbrink and Kiss, 2019), the suitability of regolith to support growth (Wamelink *et al.*, 2014; Table 1), and the role of atmospheric gases on germination (Musgrave *et al.*, 1997). Oxygen and ROS are required for the successful germination of most crop plants (Gu *et al.*, 2012). Previous studies (Kuznetsov and Hasentein, 2003; Park and Hasentein, 2016) indicate that some species can germinate in a pO_2_ as low as 5%; however, this amount of oxygen is insufficient for alleviating hypoxia-induced stress and hypoxia-associated metabolic changes.

Access to partial oxygen pressure during germination and embryo growth is absolutely required for the start of photosynthesis and continued plant growth (Morris *et al.*, 2011) and is as important as access to water (Wang *et al.*, 2015). While anoxia/hypoxia is an issue on Earth usually associated with flooding (Miro and Ismail, 2013) and can be overcome by a variety of agricultural practices, on Mars, it is a significant barrier to establishing crop plants to sustain a human colony. Although the molecular and physiological events associated with anoxia are well understood (Atwell *et al.*, 2015) as are the general mechanisms for germination in *L. sativum* as a model crop species (Morris *et al.*, 2011), there is not much that can be done with this knowledge to circumvent the requirement for oxygen during germination on Mars.

As noted in Section 1, it is of course possible to produce oxygen from the electrolysis of *in situ* water, but we feel that the oxygen-donating polymer described here offers an equally viable alternative or a system that can be used in conjunction with other oxygen-generating methods. Although size optimization experiments for the oxygen-donating polymer have not been conducted, the size range of particles (Table 2 and Fig. 2) is primarily between 600 and 1000 μm. This means that they would not be an inhalation hazard in transit. Perhaps, additional plant synthetic biology approaches can be employed to lower the pO_2_ requirement for selected crops (Llorente *et al.*, 2018) to lessen the need for *in situ* water conversion to oxygen or reducing the amount of the synthetic oxygen-donating polymer needed to support martian crop plants or bioregenerative life support systems.

On Mars, it is of course possible to provide oxygen directly to a crop enclosure via a number of sources, but these may not be completely useful or sustainable, especially considering the crop plant acreage required for even a modestly sized long-term human colony. Hence, an additional crop seed germination system may be useful, especially for establishing the first crop generation. Once crops reach levels that are sufficient to feed a colony and run biological-based life support systems, the molecular oxygen produced from the plants (Figs. 4 and 8) can theoretically serve the germination requirement for the next crop generation, depending on enclosure volume per plant. Hence, the oxygen-donating lightly cross-linked polyacrylate material may only be required for the first-generation crop planting or as a supplement for additional growing cycles. Intriguing is the possibility that reduced regolith itself may ultimately be an excellent molecular oxygen sink.

In addition to the oxygen requirement is the difficulty of martian regolith to sustain plant growth due to the little or no useful nutrient content (Table 1); however, recent studies from the Curiosity rover (Stern *et al.*, 2015) may indicate the presence of nitrate in selected locations. In contrast, organic material may have been degraded over geologic time due to the presence of oxidants in regolith; most notably, perchlorate and surface minerals that contain metal oxides (Lasne *et al.*, 2016). These oxides might be themselves deleterious to crop plant growth. Still, it is possible that regolith may offer several advantages over hydroponic and other technologies (Schuerger *et al.*, 2002). Although it is still unclear whether actual Mars regolith will successfully support sustained crop growth, it is important to identify alternative crop microenvironmental growth systems as a first step to the establishment of a sustainable Mars agricultural presence.

We have developed a polymer-based system that is capable of supporting *L. sativum* seed germination and plant growth in JSC Mars-1A regolith simulant under a pressurized (101 kPa) Mars atmosphere. The lightly cross-linked oxygen-containing polyacrylate foam appears to function both as an oxygen-releasing matrix and as a water-retaining hydrogel (Figs. 3 and 4). Analysis of the roots on harvesting shows that the roots mass around and into the lightly cross-linked polyacrylate gel particles, which help to stabilize the plant and to promote continued growth. This is the first report that we are aware of that successfully germinated and grew a plant in a fully carbon dioxide atmosphere in a Mars regolith simulant.

This advance potentially means that a significant amount of *in situ* Mars resources can be utilized to support the agriculture required to sustain a human colony. Having a system that allows for the maximal utilization of martian resources may increase the probability of permanent food crops and biological life support systems. Especially as there are emerging methods (*e.g.*, this work) to germinate seeds on Mars. The optimal concentration of the oxygen-donating lightly cross-linked polyacrylate material means that only 8 kg would be needed to fully oxygenate a metric ton of martian regolith (Fig. 8).

*L. sativum* (garden cress) was selected for these initial experiments because of its small seed size and short growth period. In addition, it is a model crop plant that has been extensively studied. The small seed size means nutrients within the seed are rapidly depleted and growth is dependent on the regolith simulant composition. The short germination/growth period means that we could utilize a minimum number of vacuum desiccators and efficiently survey a large number of growth conditions. Finally, *L. sativum* is known to successfully grow under a variety of conditions on Earth.

The lightly cross-linked polyacrylic acid, sodium salt hydrogel described here (Fig. 1), is unique due to the low level of internal cross-linking (∼3%). This produces greater mechanical flexibility and swelling characteristics during the oxygen-foaming process compared with other forms of cross-linked polyacrylates. These physical properties then facilitate quantitative oxygen release during rehydration. Initial experiments attempted oxygen foaming of commercial superabsorbent material powder (HySorb 8760AD, BASF Ludwigshafen Germany; ∼36% cross-linked). Results showed significantly less swelling and formation of a hard, solid mass that did not retain a foamed structure (data not shown). So clearly, the degree of internal cross-linking is the primary variable for oxygen release ability.

In regard to the carbon dioxide atmosphere experiments, the oxygen-containing lightly cross-linked polyacrylic foam powder appears to successfully deliver dissolved oxygen to both the water in the regolith simulant and as a gaseous oxygen diffused through the regolith and into the atmosphere above the regolith (Fig. 4). It delivers enough oxygen to initiate seedling germination and early growth of *L. sativum* seedlings (Figs. 6 and 7; Tables 3 and 4). Finally, it acts as a water reservoir and support matrix for the roots to grow through and around (Fig. 3). It is unclear at this time as to whether the polyacrylate or polyacrylate breakdown products can act as a carbon source for the seedling; this is currently under investigation.

Park and Hasenstein (2016) published a article on the oxygen requirement for *Brassica rapa* seed germination. They determined that a pO_2_ ≥10% was required for normal (vs. delayed) germination. Our results are consistent with this finding in that we observe no germination delay in a 100% CO_2_ atmosphere in the presence of the oxygen-donating lightly cross-linked polyacrylate. In addition to polymer-based or mesoscale materials approaches, nanomaterials have proven and are of interest in helping plants overcome abiotic stress including anoxia (Kahn *et al.*, 2017), so perhaps a combined approach would be fruitful.

This work was designed to answer a simple question: could a regolith additive support the germination of plants in a primarily CO_2_ atmosphere. Clearly, the results for *L. sativum* are yes. The polymer donates enough molecular oxygen to germinate 100% of seeds compared with 0% of seeds planted alone in regolith simulant or in the presence of lightly cross-linked polymer devoid of oxygen-donating ability (Table 3). The system produces enough sustained oxygen donation to support plant growth through third leaf emergence (Figs. 6 and 7; Table 4), and the increase in measured growth vessel oxygen levels indicates that photosynthesis had begun (Fig. 10). Experiments were conducted for 12 days initially because the focus was on germination and early development.

The 24-day growth period is the limit for this experimental design as the regolith simulant substantially dries out (Fig. 9). A primarily CO_2_ atmosphere was chosen for study (vs. an N_2_ or Ar atmosphere) because that is what is found on Mars. Initial experiments were conducted at 101 kPa to begin to characterize the system and in no way reflect an assumption that martian agriculture will be performed in fully pressurized enclosures. Hence, this is a first step toward creating a viable crop agriculture/bioregenerative life support system that can utilize more Mars *in situ* resources.

Although promising, there are some important questions to consider as this work progresses. The first is obtaining a better understanding of how the system behaves, specifically O_2_ movement through the regolith simulant, due to lowered convection in a reduced gravity microenvironment. We are currently adapting the growth chamber to function on a random positioning machine platform where the gravity vector can be set to 0.38 g.

Other current work entails looking at the ability of oxygen-containing lightly cross-linked polyacrylate foamed material to support germination under reduced atmospheric pressure and in the presence of a nearly exact martian atmosphere (96% CO_2_, 2% Ar, 2.0% N_2_) versus a “martian-like” atmosphere. If successful, this would alleviate the need to fully pressurize plant growth enclosures (Corey *et al.*, 1996). Plant responses to hypobaria, however, may be a key limiter in the viability of plants grown at reduced atmospheric pressure on Mars (Corey *et al.*, 2002; Richards *et al.*, 2006), especially below 40 kPa (Zhou *et al.*, 2017). The effects of reduced atmospheric pressure vary between plant species and in response to different atmospheric gas mixtures (Goto *et al.*, 2003).

In general, plants have been shown to grow in as low as 10 kPa pressure (Paul *et al.*, 2004) if the hypoxic load can be reduced (He *et al.*, 2007; He and Davies, 2014). Indeed, *Arabidopsis* seed production can be maintained at 10 kPa if adequate oxygen partial pressure is maintained (Goto *et al.*, 2002). Reduced atmospheric pressure is not always bad for plants (Richards *et al.*, 2006; Tang *et al.*, 2015), and in fact, reduced pressure environments may be well suited for martian plant growth enclosures (Paul and Ferl, 2006; Wheeler, 2010).

Also of importance as the system is further refined is to ascertain the performance of the material in different Mars regolith simulants (Schirmack *et al.*, 2015), since there is some degree of Mars regolith heterogeneity based on planet location (Meslin *et al.*, 2013) and to determine what other crops can be grown, like *L. sativum*, using this system. The oxygen-donating lightly cross-linked polyacrylate system may have applications to terrestrial agriculture by allowing successful crop formation in less hospitable soils or at higher altitudes (Bechtold, 2018; Steinbrecher and Leubner-Metzger, 2018).

Another question that may impact utilizing an *in situ* martian atmosphere for agriculture and/or bioregenerative life support systems is the effect that a mostly CO_2_ atmosphere may have on seed germination and plant growth (Kaplan *et al.*, 2012; Parmesan and Hanley, 2015; Kimball, 2016). Elevated atmospheric CO_2_ reduces stomatal density, stomatal index, and stomatal conductance, all of which result in reduced transpiration (Xu *et al.*, 2016). Inside leaves, an elevated CO_2_ level increases photosynthesis, starch accumulation, water use efficiency, and PSII photochemistry efficiency (Habermann *et al.*, 2019).

Elevated CO_2_ also has effects on a variety of cellular and growth processes (Xu, 2015; Gamage *et al.*, 2018; Zheng *et al.*, 2019). Some of this effect can be ameliorated with slight increases in temperature (Britto de Assis Prado *et al.*, 2016; Habermann, *et al.*, 2019), by altering the enzymes involved in phloem loading (Ainsworth and Lemonnier, 2018), by increasing light intensity (Pan *et al.*, 2019), and by terrestrial adaptation of important crop plants such as soybean *Glycine max* (Bishop *et al.*, 2015).

A roadmap for genetic targets to improve soybean productivity has been presented (Ainsworth *et al.*, 2012), many of which could result in lessening the impact of elevated CO_2_ on plant germination and growth. In the radish, the effects of CO_2_ are worse when combined with hypobaria (Gohil *et al.*, 2010). The effect of elevated CO_2_ varies between C_3_ and C_4_ plants, although there are some exceptions such as the C_4_ plant millet (Li *et al.*, 2019), which behaves more like a C_3_ plant. The impact of elevated CO_2_ also varies in intensity between species and the published effects of elevated CO_2_ are both positive and negative.

For instance, in grains, elevated CO_2_ produces an increase in above ground plant mass and grain yield, while at the same time, it decreases nitrogen, free amino acid concentration, and protein accumulation (Soba *et al.*, 2019). Reduced stomatal density in wheat under elevated CO_2_ conditions also results in increased water use efficiency (Dunn *et al.*, 2019), as is seen in sorghum (Wall *et al.*, 2001), while a study (Leakey *et al.*, 2006) indicated little effect of elevated CO_2_ on corn.

In rice, higher CO_2_ levels improve phosphorus utilization that is directed toward plant growth (Zhu *et al.*, 2019) and in *Arabidopsis thaliana*, elevated CO_2_ has been shown to reduce oxidative stress (Abo Gamar *et al.*, 2019), but probably not through alterations to antioxidant defenses (AbdElgawad *et al.*, 2016). In fact, two recent reviews (Dong *et al.*, 2018; Uddling *et al.*, 2018) discuss increases in yield, but decreased nutritional quality in vegetables grown in elevated CO_2_ atmospheres. So, the cellular and physiological impact of germination and growth at the elevated levels of CO_2_ in the cited references (typically around 700 ppm) is complex.

A clear area of study needed to advance this work is to determine the cellular and physiological effects that a greatly elevated (>700 ppm) CO_2_ concentration has on plant germination, development, and growth (for a variety of potential crop/bioregenerative life support system species). In addition, the degree of regolith simulant acidification (and its impact on plant growth) caused by a Mars-like level of CO_2_ needs to be elucidated.

There are also questions concerning how best to scale up this system. The current oxygen-donating lightly cross-linked polyacrylate is a viable component of a holistic approach to crop and regenerative life support systems that include multiple methods of martian water extraction (Ralphs *et al.*, 2015), synthetic biology approaches to crop and resource management (Menezes *et al.*, 2015; Llorente *et al.*, 2018), the overall design of bioregenerative life support systems (Fu *et al.*, 2016), and novel habitat design/construction (Rothschild, 2016). At this time, the material can be formulated and produced at the 100 kg scale, with no impediments to production at greater quantities.

In conclusion, the oxygen-donating lightly cross-linked polyacrylate material is capable of supporting germination in an otherwise oxygen-free atmosphere. Germination rates were 100% for the oxygen-donating material versus zero percent for the nondonating material. In addition, the system produced sufficient O_2_ to support *L. sativum* development and growth through the onset of photosynthesis/transpiration. Hence, the oxygen-donating lightly cross-linked polyacrylate material may represent a valuable addition to the tools and techniques available to design agricultural systems that utilize as many *in situ* Mars resources as possible and in the design of bioregenerable life support systems to help maintain a martian colony.

## Supplementary Material

Supplemental data

## Abbreviation Used

ROS: reactive oxygen species
